# Epithelial-mesenchymal transition and senescence: two cancer-related processes are crossing paths

**DOI:** 10.18632/aging.100209

**Published:** 2010-10-17

**Authors:** Marjon A. Smit, Daniel S. Peeper

**Affiliations:** Division of Molecular Genetics, The Netherlands Cancer Institute, 1066 CX Amsterdam, The Netherlands

**Keywords:** EMT, senescence, cancer, Twist, Zeb1

## Abstract

The epithelial-mesenchymal transition is involved in several physiological processes. However, it is also believed to contribute to cancer progression. Conversely, cellular senescence constitutes a failsafe program against cancer progression. Interestingly, EMT and senescence seem to cross paths, with several factors playing dominant roles in both settings. Here, we describe recent observations that link these important cellular processes.

## Epithelial-mesenchymal transition

Cancer is a complex disease against which mammals have developed multiple protective mechanisms. Late-stage cancer is almost invariably accompanied by metastasis, accounting for the most common cause of death of cancer patients. The metastatic cascade comprises several steps, ultimately leading to the emergence of secondary tumors at distant sites from the primary lesions [1]. One process contributing to the first phase of metastasis is the epithelial-mesenchymal transition (EMT). EMT is well known for its important roles in embryogenesis and development, in which epithelial cells acquire properties reminiscent of those of mesenchymal cells. Full differentiation and the establishment of a specific tissue architecture may involve several rounds of EMT, but also of the reverse process, mesenchymal-epithelial transition (MET) [2].

EMT is accompanied by the loss of the cell-cell contacts so typical of epithelial cells, and the acquisition of migratory and motile properties. It is for these reasons that EMT can have adverse effects to the organism, contributing to pathological processes such as fibrosis and cancer [3]. In particular, when adopted by cancer cells, EMT allows for the invasion and intravasation of tumor cells into surrounding tissues, blood and lymphatic circulation [4,5]. Similar to its physiological role, also in metastasis EMT conceivably corresponds to a transient and dynamic process, which involves several steps. Furthermore, it does not appear to occur in the bulk of the tumor cells but rather locally, at the invasive fronts of a tumor [6-8].

Epithelia are formed by polarized layers of epithelial cells, which are tightly connected by adherens junctions. These consist of E-cadherin, α-, β- and γ-catenin and are connected to the actin cytoskeleton, thus providing the cells with a rigid structure. During EMT, several epithelial proteins, like E-cadherin, α-, β- and γ-catenin are downregulated, whereas mesenchymal proteins, including vimentin, fibronectin and smooth muscle actin, can be upregulated [9]. The most common characteristic of EMT is the downregulation of E-cadherin. This glycoprotein can be repressed by two types of transcription factors, basic helix-loop-helix transcription factors including E12/E47, Twist1 (Twist) and Twist2 (Dermo1), and the zinc finger transcription factors, including the Snail family (Snail or Snai1 and Slug or Snai2), and the Zeb family, comprising Zeb1 and Zeb2 (also called SIP1) [10-16]. These transcription factors bind to E-boxes within the *CDH1* promoter and thereby suppress its transcription. Most of these factors have been demonstrated to play a critical role in invasion and metastasis [11,16-19]. EMT can be induced by several oncogenes, including RAS^V12^ [20], ErbB2 [21] and TrkB [22,23]. They activate multiple effectors including the PI3 and MAP kinases, but also the Wnt, Notch and NFkB pathways, all of which are involved in EMT regulation [24-26], can be activated.

## Cellular senescence

Another mechanism involved in cancer progression is cellular senescence. Senescent cells fail to proliferate, but remain metabolically active. Senescence can be triggered by short or malfunctioning telomeres (called replicative senescence), but also prematurely, by a variety of stress signals, including unscheduled oncogenic signaling [27,28]. “Oncogene-induced senescence” (OIS), as the latter phenomenon is called, relies on the activation of tumor suppressor gene networks often comprising RB and p53, mediating cell cycle arrest [29]. In addition to elevated tumor suppressor signaling, OIS is associated with several other hallmarks, including increased activity of lysosomal β-galactosidase (SA-β-GAL), chromatin remodeling and induction of DNA damage [30]. In many settings, OIS is associated with the secretion of dozens of cytokines, comprising the “Senescence-Messaging Secretome”, or SMS, denoting its communicative role [31]. Cellular senescence can be triggered not only by oncogene activation, but also by the loss of tumor suppressor genes, including *PTEN*, *NF1* and *RB* [32].

Although OIS has long been viewed as an exclusive *in vitro* phenomenon, it is being increasingly recognized as a critical feature of mammalian cells to suppress tumorigenesis, acting alongside cell death programs like apoptosis. For example, human melanocytic nevi (moles), benign tumors that have a low propensity to progress towards melanoma, display several hallmarks of OIS. In addition to harboring an oncogenic mutation (most commonly BRAF^E600^), they display little proliferative activity, a characteristic that is typically maintained for decades. Furthermore, nevi express elevated levels of p16^INK4A^ and have increased SA-β-GAL activity [33,34]. Similar results have recently been reported for BRAF^E600^ knockin mice in which the activated kinase is expressed selectively in the melanocytic compartment [35,36]. Several additional mouse models have also shown senescence biomarkers in early neoplastic lesions[37]. For example, lung adenomas expressing oncogenic RAS^V12^ are in a senescent state in contrast to invasive adenocarcinomas that are proliferating [38].

## EMT players regulating senescence

For almost two decades, the transcription factor Twist has been known for its important role in embryonic development [39]. More recently, Twist, but also other EMT regulators, have attracted considerable attention for their contribution to cancer progression. For example, Weinberg and co-workers found that Twist induces EMT and, as such, plays a critical role in metastasis [11]. Other transcription factors from the Snail and Zeb family are endowed with similar capacities [40]. Earlier, a role for Twist 1 and 2 in apoptosis was revealed in an expression library screen for cDNAs suppressing the pro-apoptotic effect of the *myc* oncogene in MEFs [41]. In that setting, Twist reverts p53-induced cell cycle arrest and suppresses *Arf*, a gene that is highly induced during cellular and oncogene-induced senescence [42,43]. Similarly, in human prostate epithelial cells, Twist bypasses cellular senescence in an p14^ARF^-dependent fashion [44]. Conversely, RNAi depletion of Twist induces cell cycle arrest in gastric cancer cells, which correlates with regulation of the major ARF effector, p53 [45].

Further mechanistic insight into how Twist impacts on the cell cycle machinery has evolved. Twist over-expression prevents the upregulation of p21^CIP1^ and p53 upon genotoxic stress in several cell lines [46-48]. In addition to indirect regulation through ARF, Twist may regulate p53 by a direct interaction, thereby preventing it from binding to DNA [49]. Consistent with this, adriamycin treatment leads to increased levels of a protein complex comprising p53, MDM2 and Twist [47]. A search for upstream targets revealed that PKB/AKT can phosphorylate Twist at Ser42, inhibiting p53 activity in response to γ-irradiation and promoting cell cycle progression [48]. Ansieau *et al.* showed that Twist affects the transcriptional regulation of p16^INK4A^ and p21^CIP1^ [50], arguing together with previous publications that Twist can simultaneously deregulate the p53 and RB pathways, both of which affect several processes, including senescence. The signaling pathways targeted by Twist may even go beyond this, as suggested by the observation that Twist enhances the transforming activity of N-MYC in *Ink4a-ARF^-/-^* MEFs [46]. Twist can also augment the transforming effects of E1A and Ras^V12^ [41], although it remains to be seen whether Twist acts in such contexts during human tumor progression [51].

Extending these findings, increasing evidence suggests that the two processes that seem to operate independently, EMT and senescence, are in fact intertwined. For example, while RAS^V12^ induces EMT in epithelial cells, often in a cooperative fashion with TGFβ[20], it also induces senescence in human diploid fibroblasts [27]. Puisieux and co-workers showed that whereas ectopically expressed ErbB2 induces senescence, overexpression of both Twist and ErbB2 triggers EMT and allows for senescence bypass, both in MEFs and human epithelial cells [50]. This is consistent with the prevailing view that a single oncogene is insufficient to drive cancer progression: it commonly acts cytostatically or induces a death program and requires a collaborating oncogene to convert this into a pro-survival and mitogenic process [37]. But more importantly, the studies mentioned above, and several that will be discussed below, have provided a link between EMT and cellular senescence.

The premise that activation of EMT is linked to suppression of cellular senescence has been proposed also in the context of another EMT regulator, Zeb1 [52]. Specifically, depletion of *Zeb1* in MEFs causes MET, which is characterized by increased expression of epithelial proteins such as E-cadherin and decreased expression of mesenchymal proteins. Zeb1 loss triggers premature senescence by binding directly to the *CDKN1A* and *INK4B* promoters, thereby simultaneously affecting p53 and RB signaling. In turn, Zeb1 is regulated via the Zeb1/miR200 feedback loop, which is thought to drive cancer progression via promoting EMT and inhibiting senescence [53].

These findings prompt several interesting questions. Probably most importantly, does the coordinated deregulation of EMT and senescence reflect only a “collateral effect”, or instead, are there mechanistic links that tie these two processes together [54]? A recent study suggests the latter possibility [55]. Esophageal squamous cell carcinoma cells expressing activated EGFR were shown to undergo premature senescence. A subpopulation of cells emerged from this pool, which expressed elevated levels of Zeb1 and 2, as if these factors suppressed the senescence program. Interestingly, when cells were locked in a senescent state by activation of p53, the cytokine TGFβ was no longer able to induce EMT, raising the possibility that senescent cells cannot undergo EMT. This is in line with the findings of Ansieau *et al*. that senescence abrogation is accompanied by an EMT [56].

Although a general role for other EMT-associated transcription factors in senescence remains to be elucidated, some can regulate several cell cycle regulators that have also been implicated in senescence. For example, in MDCK canine epithelial cells, Snail deregulates the cell cycle machinery by induction of p21^CIP1^ and reduction of cyclin D1 and D2, thereby effectively inducing cell cycle arrest [57]. Also in HEPG2 cells, Snail induces cell cycle arrest via regulation of p15^INK4B^ [58], a relative of p16^INK4A^ that has also been associated with senescence [59]. Conversely, in combination with an oncogene, Snail can have the opposite effect, for example by inhibiting E2A-induced p21^CIP^ induction [60]. Furthermore, Snail can suppress p53 by direct binding, at least in certain settings [61,62]. Overexpression of another transcription factor, Zeb2, reduces the proliferative capacity of cells, involving inhibition of cyclin D1 [63]. However, although from these results it appears that EMT-associated transcription factors can regulate the cell cycle through proteins that are also involved in senescence, it remains to be established whether these modes of communication, indeed, have an impact on senescence signaling.

## Senescence players regulating EMT

Whereas several prototypic EMT regulators have been implicated in senescence, conversely, a number of key senescence players have been found to affect EMT. For example, the viral oncoprotein SV40 LT, which simultaneously deregulates the cell cycle regulators p53 and RB, can suppress E-cadherin to induce a mesenchymal-like morphology. This is dependent on the downregulation of RB [64]. As RB was recently demonstrated to play a unique role, among the pocket protein family, in cellular senescence [65], these findings may link RB, senescence and EMT. Also in another setting, when EMT is induced by TGFβ/TNFα in MCF10A cells, RB is downregulated. Furthermore, whereasoverexpression of RB blocks the morphological effect and the suppression of E-cadherin expression [66], downregulation of RB induces EMT [67]. In both cases this is regulated by binding of RB to the *CDH1* promoter, thereby suppressing its transcription [66,67]. A link between RB and E-cadherin was also described in a slightly different setting: E-cadherin-mediated aggregation prevents cell death induced by active PKC. This is accompanied by RB activation, cell cycle arrest and survival [68].

Wild type p53, a key senescence player [28], inhibits the transcription factor Slug (or Snai2) via MDM2, whereas mutant p53 stabilizes Slug, thereby increasing its levels and enhancing cancer cell invasiveness [69]. In non-small-cell lung cancer, where p53 is often mutated, Slug expression is high, correlating with low MDM2 levels and lower overall survival.

Another factor that can be associated with cellular senescence and has also been implicated in EMT is p21^CIP1^. It was shown that p21^CIP1^ is inhibited during Ras^V12^-induced EMT in MCF10A cells. Furthermore, whereas transgenic mice expressing Ras^V12^ display only some features of EMT, compound transgenic mice with a deficiency for p21^CIP1^ show accelerated mammary tumor formation, which may result from the induction of more extensive EMT features [70]. This suggests that p21^CIP1^ plays a role in EMT both not only *in vitro* but also *in vivo*. But again, to what extent this CKI causally connects senescence and EMT remains to be seen.

The inflammatory protein network seems to represent yet another link between EMT and senescence. It has recently been shown that OIS is accompanied by the induction of several interleukins, revealing that senescence is commonly associated with a robust inflammatory response [31,71,72,73]. Unexpectedly, OIS can be abrogated (or even reverted) by downregulation of either interleukin 6 (IL-6) or IL-8 [71]. The latter is specifically co-expressed with p16^INK4A^-positive non-proliferating cells in colon adenomas, suggesting that some interleukins contribute to tumor suppression in benign human lesions [71]. Similarly, silencing of a chemokine receptor, CXCR2, results in bypass from senescence [72]. On the other hand, IL-6 is able to induce EMT in epithelial cells, suggesting that interleukins also participate in the regulation of EMT [74]. Furthermore, EGF-induced EMT in ovarian carcinoma cells induces both IL-6 expression and secretion [75]. Also IL-8 is associated with EMT: it is induced in cells undergoing TGFβ-induced EMT [76]. Interestingly, CXCR1 (but not CXCR2) is also induced in these cells. The interleukin-related protein ILEI was shown to be important for EMT and metastasis [77]. These observations suggest that the inflammatory network is important for both senescence and EMT.

## Concluding remarks

**Figure 1.**
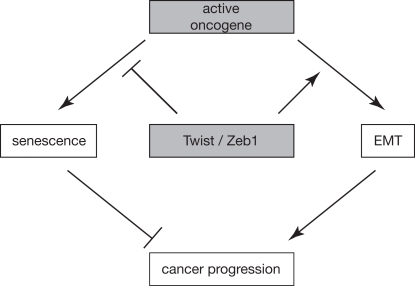
Working model schematically depicting how EMT and senescence are linked and contribute to cancer progression. An active oncogene can either induce senescence or EMT, dependent on the cellular context. Conversely, transcription factors like Twist and Zeb1 can have a double impact on cancer progression by simultaneously inhibiting oncogene-induced senescence and promoting EMT.

Recent observations suggest that two important processes involved in cancer progression, senescence and EMT, are crossing paths. For example, several transcription factors can both inhibit senescence and induce EMT (Figure 1). In doing so, they seem to have a double role in promoting cancer: while an override of the senescence program contributes to the acquisition of indefinite proliferative capacity, induction of EMT bypasses rate-limiting aspects of the metastatic cascade. Conversely, at least in theory, this may open new strategic therapeutic avenues that, by targeting the major players promoting EMT and senescence, have a double impact. This potential perspective not withstanding, there are several important questions remaining. Although senescence bypass can be accompanied by EMT, it will be of interest to determine whether, in fact, EMT contributes to the override of senescence. Indeed, although several observations have revealed features of EMT in association with (abrogation of) cellular senescence, or vice versa, in many cases it remains to be established whether the processes are mechanistically tied together. For example, is there a direct role for E-cadherin in the regulation of senescence? While Twist and other transcription factors that can promote EMT have multiple effector genes, also several senescence players, including *CDKN2A*, harbor E-boxes in their promoters [78], suggesting that the EMT-associated transcription factors can also regulate the cell cycle without direct regulation of E-cadherin. Finally, it is interesting to note that EMT and senescence share the phenomenon that the biomarkers that accompany these processes are regulated differentially in different (genetic and cellular) settings, for reasons that are as yet largely unclear. Answers to these and related questions will not only increase our understanding of these two mechanisms driving cancer progression, but eventually also help to improve strategies for therapeutic intervention.
